# Association of menarche age with macrosomia and modified effect from dietary pattern: findings from the Chinese pregnant women

**DOI:** 10.3389/fnut.2026.1777526

**Published:** 2026-04-02

**Authors:** Yuan Jin, Lan Zhang, Qian Ma, Xinyu Zhang, Aohua Liu, Xiang Li, Siyang Chen, Shunming Zhang, Jing Lin, Wei Zhang, Le Ma

**Affiliations:** 1 General Practice Department, The First Affiliated Hospital of Xi'an Jiaotong University, Xi'an, China; 2 School of Public Health, Xi'an Jiaotong University Health Science Center, Xi'an, China; 3 Key Laboratory for Disease Prevention and Control and Health Promotion of Shaanxi Province, Xi'an Jiaotong University, Xi'an, China

**Keywords:** age at menarche, cohort study, dietary pattern, macrosomia, modified effect

## Abstract

**Background:**

Age at menarche (AAM) is increasingly identified as essential factor of various perinatal complications. However, evidence on AAM with incident macrosomia remains to be determined, as does the potential modifying effect of behavioral factors. This study aimed to examine the prospective association between earlier AAM and development of macrosomia and to evaluate whether dietary patterns modify this association.

**Methods:**

This prospective cohort study comprised 2,554 Chinese women from Xi'an Birth Cohort. AAM was ascertained by self-report. Dietary intake was evaluated using a validated semi-quantitative food frequency questionnaire, and principal component analysis was used to derive three dietary patterns, named “meat, shrimp, and fish,” “vegetables, soybean and soybean products” and “animal offal, processed meat, and baked food.” Binary logistic regression models were used to estimate odds ratios (ORs) and 95% confidence intervals (CIs) for risk of macrosomia associated with AAM and dietary patterns. The non-linear relationships between AAM and macrosomia were explored by restricted cubic splines. Stratified analyses were conducted to examine the roles of dietary patterns.

**Results:**

Compared with women experienced menarche between 13 and 15 years, women with menarche before age 13 years had a higher risk of macrosomia (OR: 1.90; 95% CI: 1.37–2.62, *P* for non-linear < 0.05). Adhering to the “meat, shrimp, and fish” dietary pattern exhibited a reduced risk of macrosomia (OR: 0.64, 95% CI: 0.46–0.89). Similarly, following the “vegetables, soybeans, and soybean products” dietary pattern demonstrated a 52% reduction in the risk of macrosomia (OR: 0.48, 95%CI: 0.33–0.70). But the processed food dietary pattern increased the risk by 1.23 times (OR: 2.23, 95% CI: 1.42–3.50). Dietary may influence the odds of macrosomia in earlier AAM.

**Conclusions:**

Earlier AAM is associated with higher risk of macrosomia. This risk may be attenuated by following a vegetables and soy products diet model but exacerbated by adhered to a processed foods diet. These findings highlight the potential for dietary habits to modify for macrosomia, particularly among women with early menarche.

## Graphical Abstract

**Figure:**
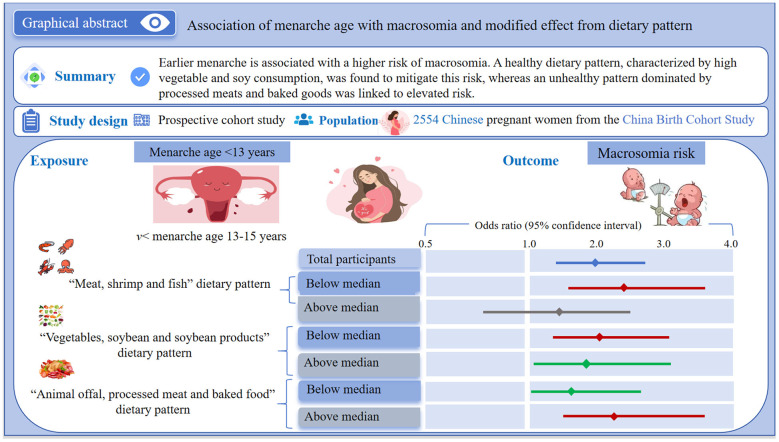

## Highlights

- A study investigated the association between AAM and the risk of macrosomia.
- A healthy diet rich in vegetables and soy products associated with reduced risk of macrosomia in the context of established maternal health implications of early AAM.
- While an unhealthy diet dominated by processed meats and baked goods increased risk of macrosomia.
- These findings provide critical epidemiological evidence for the importance of pre-pregnancy diet in macrosomia prevention.

## 1 Introduction

Macrosomia, defined as an absolute birth weight exceeding 4,000 grams irrespective of gestational age (1), represents a significant obstetric and public health concern with a global prevalence ranging from 0.21% to 9.2% (2–4). A growing body of evidence indicates that this condition is associated with spectrum of adverse perinatal outcomes, such as postpartum hemorrhage and infection, as well as fetal and neonatal risks, including shoulder dystocia, perinatal asphyxia, hypoglycemia, and fetal demise (5–8). Moreover, it would increase risks of cardiovascular diseases in later life (9–11). Furthermore, a pilot evaluation estimate suggested that neonatal complications of a macrosomia lead heavy economic burden (12). These risks show the critical need for early identification of at-risk women and the development of effective strategies for macrosomia prevention.

Age at menarche (AAM), marking the onset of menstruation in female puberty, is a critical indicator of sexual development. Previous research has indicated that genetic loci associated with earlier AAM increased offspring birth weight (13). Additionally, early AAM seemed associated with hormonal alterations, metabolic dysregulation, and inflammatory (14–20), pathophysiological changes that are critical mechanisms underlying the incidence of macrosomia (21, 22). However, the potential association between early AAM and the macrosomia remains inconclusive. While lifestyle factors, particularly diet, have been hypothesized to modulate macrosomia risk through hormonal, metabolic and inflammatory factors (21, 23), existing evidence is inconsistent (24, 25). This inconsistency may be attributed to several methodological limitations, including small sample sizes, heterogeneous study designs, as well as the dietary intervention studies have primarily focused on parous women with single dietary nutrient interventions. In addition, the complexity of dietary impacts on health outcomes underscores the necessity for a comprehensive approach to dietary assessment (26). Therefore, the analysis of dietary patterns is crucial for elucidating the association between diet and macrosomia, particularly regarding the extent to which specific dietary patterns may modify the association between early AAM and macrosomia risk.

This prospective cohort study from the Xi'an Birth Cohort study (XBC) aimed to explore the association between AAM and macrosomia and elucidate the potential roles of dietary pattern in modulating this association.

## 2 Material and methods

### 2.1 Study population

XBC was conducted to explore the associations between maternal lifestyle, nutrition, environment exposure and perinatal health outcomes among a population in northwest China. The study design and methodological framework have been described in previous publications (27, 28). Briefly, pregnant women aged 18 to 49 years were enrolled at 6–16 weeks' gestation during an antenatal care between April 2021 and September 2023. In this study, we included pregnant women aged 18 to 49 years, who were capable of independently completing relevant questionnaires in Chinese. The exclusion criteria included pregnant women who did not respond to the initial maternal interview (*n* = 738), those with serious heart, liver, or kidney disease (*n* = 97), non-single pregnancy (*n* = 365), and those with extreme total energy intake (< 500 kcal/day or >3500 kcal/day) (*n* = 158). A total of 2707 women were followed, with 2554 included in the final analysis (Supplementary Figure 1).

The XBC study protocol was reviewed and approved by the Medical Ethics Committee of the Health Center at Xi'an Jiaotong University (Approval No. 2020-1263). All participants provided written informed consent prior to their inclusion in the research.

### 2.2 Age at menarche

AAM was assessed via an open-ended question in the pregnancy history questionnaire which participants completed 24 h after enrollment: “How old were you when you first experienced menstruation?”. Referring to the classification method established by previous studies (14, 29, 30), menarche timing was classified into three categories using AAM data: early (< 13 y), normal (13–15 y, reference), and late (>15 y).

### 2.3 Dietary intake

Dietary intake was evaluated through a 108 items semi-quantitative food frequency questionnaire (FFQ), designed for pregnant women in rural western China, which has been documented to have acceptable reproducibility and validity coefficients ranging from 0.40 to 0.80 (31, 32). The food atlas was used to assist participants in recalling their food portions consumed over the preceding year. The frequency of consumption for each food item was categorized into nine levels. To determine the average daily intake of each food item, we employed a reference of one portion per day, adjusted by varying weight coefficients, and multiplied by the respective quantity (in grams) consumed (33).

### 2.4 Dietary patterns

Dietary patterns were identified using Principal Component Analysis (PCA) with orthogonal rotation (varimax) based on 23 food groups categorized *a priori* according to nutritional similarity and cultural consumption patterns (Supplementary Table 1). The intake for each food group was calculated by summing the consumption of individual food items, with all values standardized to grams per day. Prior to identifying dietary patterns, the suitability of PCA was confirmed through Kaiser-Meyer-Olkin (KMO) measure and Bartlett's test of sphericity (KMO = 0.769, *P* < 0.05), leading to the identification of three distinct dietary patterns (Supplementary Table 2). The factor loadings for each identified dietary pattern are detailed in Supplementary Table 3. The relative contributions of food groups to these patterns are depicted visually in the radar chart shown in Supplementary Figure 2. The factor loadings, with their absolute values indicating the contribution magnitude of each food group, were used to name the dietary patterns according to the highest-loading food items and relevant nutritional context. In this study, three dietary pattern named “meat, shrimp, and fish,” “vegetables, soybean and soybean products” and “animal offal, processed meat, and baked food,” respectively. For each pattern, a composite score was calculated by summing the standardized intake (g/day) of each food group multiplied by its respective factor loading from the principal component analysis. For Subsequent analyses, it was dichotomized based on its median value.

### 2.5 Macrosomia

Neonatal weight was measured immediately following delivery on calibrated electronic pediatric scales to the nearest 1 gram. To enhance the reliability of these measurements, standardized clinical examinations were conducted by study physicians blinded to maternal dietary intake and other potential confounding factors. In accordance with the American College of Obstetricians and Gynecologists guidelines, macrosomia was defined as a birth weight exceeding 4000 grams irrespective of gestational age (34, 35).

### 2.6 Covariates

A directed acyclic graph (DAG) was used to identify potential confounders between AAM and macrosomia (Supplementary Figure 3) (36, 37). The following covariates were selected for adjustment including age, educational attainment, employment status, monthly incomes, ethnicity, current smoking status, exposure to second-hand smoke, alcohol consumption, physical activity, parity, timing of delivery, menstrual cycle, menses length, regular menstruation, dysmenorrhea, pre-pregnancy body mass index (BMI), total energy intake, nutritional supplements usage, and dietary patterns. Physical activity was assessed using the short-form International Physical Activity Questionnaire (IPAQ).

### 2.7 Statistical analysis

To elucidate the associations between early AAM and the risk of macrosomia, we used binominal logistic regression model was to estimate the odds ratios (ORs). The median value of each AAM group was assigned as a continuous variable to test for linear trends. To evaluate the non-linear relationship, we fitted a restricted cubic spline (RCS) model with knots placed at the 10th, 50th, and 90th. Stratified analyses by key covariates were conducted to examine potential effect modification across population subgroups. Given that the associations identified in our analyses could potentially be influenced by unmeasured confounders, such as gestational diabetes mellitus (GDM) and gestational weight gain, E-values were computed to quantify the potential impact of such confounding factors. A higher E-value suggests that the findings are less likely to be explained solely by unmeasured covariates (38, 39). In addition, to estimate the robustness of our findings, we fitted a fully adjusted model excluding participants with low birth weight (< 2500 g) and those with premature off-spring (with a gestational age of at least 37 weeks).

Statistical analyses were conducted utilizing R software (version 4.3.1; R Development Core Team). A *P*- value of less than 0.05 was statistical significance.

## 3 Results

### 3.1 Participant characteristics

The baseline characteristics stratified by AAM of the study participants are presented in Table 1. Among the 2,554 pregnant women, 1,433 (56.1%) reported an AAM of 13–15 years, 961 (37.6%) reported an AAM of less than 13 years, and 160 (6.3%) reported an AAM greater than 15 years. The characteristics of the study participants were stratified by macrosomia status, with 180 cases (7.0%) identified during the follow-up period (Supplementary Table 4).

**Table 1:** Baseline characteristics of the participants according to age at menarche.

Characteristics	Total	Age at menarche (years)		
		13–15	<13	>15
Number of participants	2,554	1,433	961	160
Age (years)	30.0 (28.0, 32.0)	30.0 (27.0, 32.0)	30.0 (28.0, 32.0)	30.0 (28.0, 32.0)
Educational attainment (under college)	341 (13.4)	208 (14.5)	102 (10.6)	31 (19.4)
Employment status (no)	831 (32.5)	469 (32.7)	301 (31.3)	61 (38.1)
Monthly incomes (≤9000 CNY)	2,483 (97.2)	1,401 (97.8)	928 (96.6)	154 (96.3)
Ethnicity (minority)	25 (1.0)	8 (0.6)	14 (1.5)	3 (1.9)
Current smoker	312 (12.2)	191 (13.3)	105 (10.9)	16 (10)
Exposure to second-hand smoke	33 (1.3)	20 (1.4)	11 (1.1)	2 (1.3)
Alcohol consumption	16 (0.6)	11 (0.8)	3 (0.3)	2 (1.3)
Physical activity (MET-min/week)	600.0 (462.0, 1,158.0)	600.0 (462.0, 1,181.0)	622.0 (462.0, 1,158.0)	643.0 (495.0, 1,156.0)
Pre-pregnancy BMI (kg/m^2^)				
< 18.5	305 (11.9)	195 (13.6)	76 (7.9)	34 (21.3)
18.5–24.0	1,781 (69.7)	1,006 (70.2)	664 (69.1)	111 (69.4)
≥24.0	468 (18.3)	232 (16.2)	221 (23.0)	15 (9.4)
Parity (Primigravida)	1,204 (47.1)	692 (48.3)	444 (46.2)	68 (42.5)
Timing of delivery (weeks)	39.0 (38.0, 40.0)	39.0 (38.0, 40.0)	39.0 (38.0, 40.0)	39.0 (38.0, 40.0)
Menstrual cycle (d)	30.0 (28.0, 30.0)	29.0 (28.0, 30.0)	30.0 (28.0, 30.0)	30.0 (28.0, 30.0)
Menses length (d)	5.0 (5.0, 6.0)	5.0 (5.0, 6.0)	5.0 (4.0, 6.0)	5.0 (5.0, 7.0)
Regular menstruation (no)	173 (6.8)	93 (6.5)	66 (6.9)	14 (8.8)
Dysmenorrhea	1,235 (48.4)	713 (49.8)	447 (46.5)	75 (46.9)
Total energy intake (kcal/d)	2,358.6 (1,929.9, 2,771.8)	2,359.1 (1,922.9, 2785.7)	2,355.9 (1,923.5, 2,755.2)	2,384.8 (2,003.1, 2,785.5)
Nutritional supplements usage (no)	124 (4.9)	67 (4.7)	37 (3.9)	20 (12.5)
Macrosomia	180 (7.0)	77 (5.4)	93 (9.7)	10 (6.3)

Continuous variables were expressed as median (interquartile range) and categorical variables as n (%). BMI, body mass index; MET, metabolic equivalent.

### 3.2 Association between AAM and macrosomia

**Figure 1:**
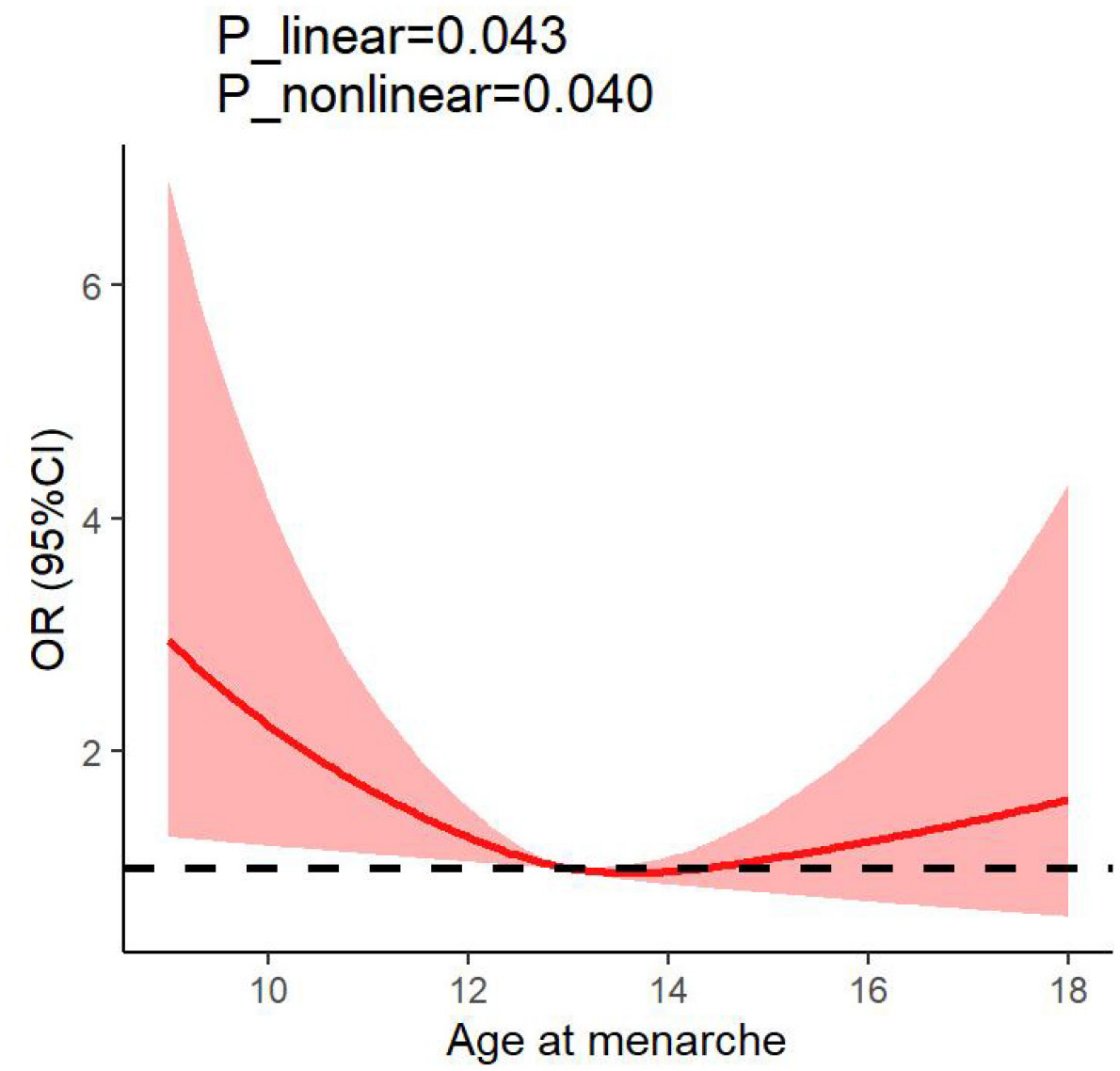
The dose-response relationships of the age at menarche with macrosomia. The solid red line describes ORs, and the rose-pink areas show 95% CIs. The logistic models were adjusted for age, educational attainment, employment status, monthly incomes, ethnicity, current smoking, exposure to second-hand smoke, alcohol consumption, physical activity, pre-pregnancy body mass index, parity, timing of delivery, menstrual cycle, menses length, regular menstruation, dysmenorrhea, total energy intake, nutritional supplements usage, and dietary patterns. Dietary patterns included “meat, shrimp and fish” dietary pattern, “vegetables, soybean and soybean products” dietary pattern and “animal offal, processed meat and baked food” dietary pattern. Three knots were placed at the 10th, 50th, and 90th percentiles of the age at menarche. CI, confidence interval; OR, odds ratio.

**Figure 2:**
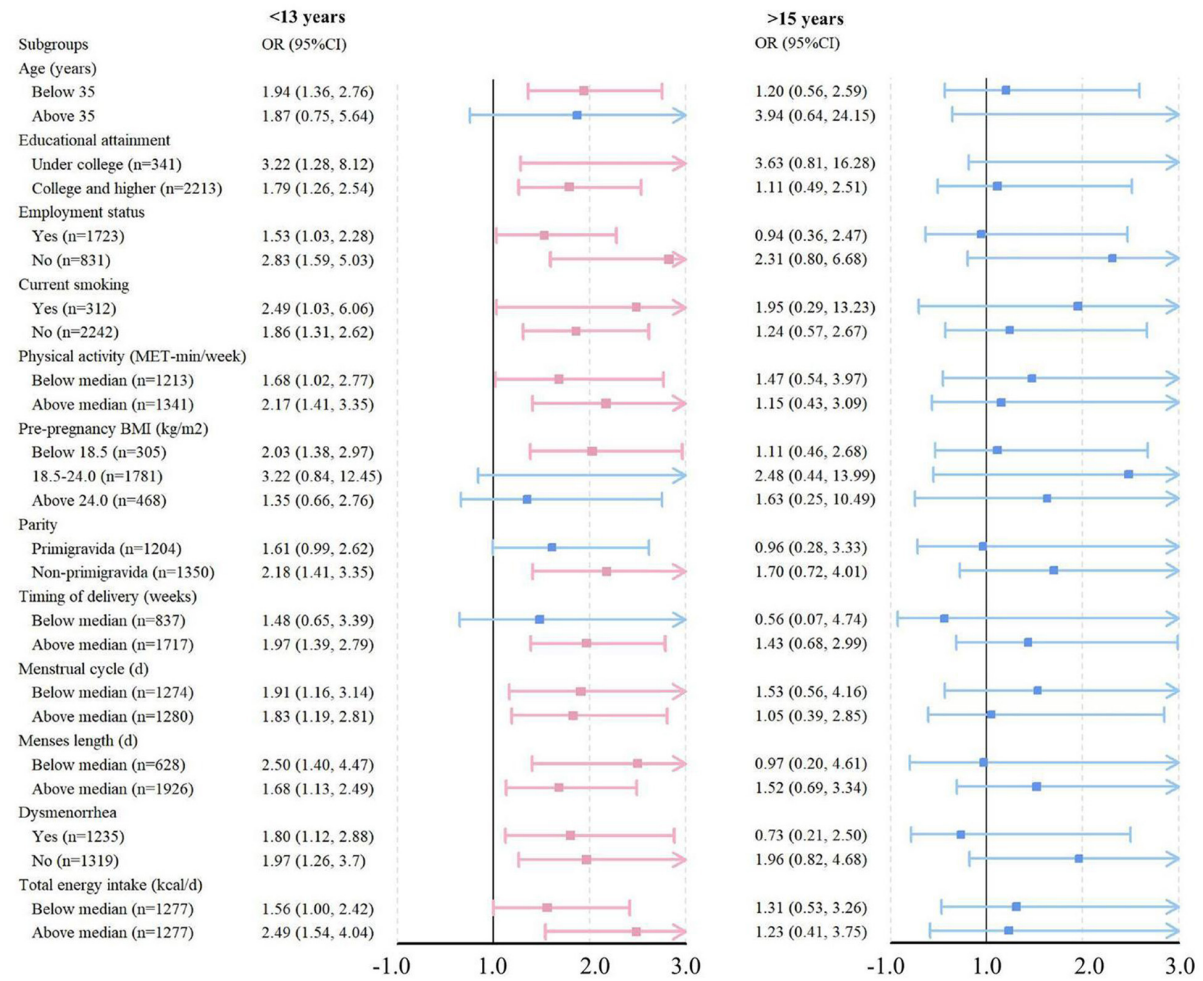
Subgroup analyses for the association of the age at menarche with the risk of macrosomia. Subgroup analyses defined by monthly incomes, ethnicity, exposure to second-hand smoke, alcohol consumption, regular menstruation and nutritional supplements usage were not conducted due to the very small sample sizes. The black vertical line represents that the OR is 1, Square indicates the point estimate of the OR, the horizontal line indicates the corresponding 95% CI, blue indicates no statistically significant difference, and pink shows that the difference is significant. With the group of women who has an age at menarche between 13 and 15 years set as the control, the left column displays the risk of delivery a macrosomia among women whose menarche age was less than 13 years. The right column exhibits the risk of giving birth to a macrosomia among women whose menarche age exceeded 15 years. Multivariable binary logistic analyses were conducted by adjusting for age, educational attainment, employment status, monthly incomes, ethnicity, current smoking, exposure to second-hand smoke, alcohol consumption, physical activity, pre-pregnancy body mass index, parity, timing of delivery, menstrual cycle, menses length, regular menstruation, dysmenorrhea, total energy intake, nutritional supplements usage and dietary patterns. CI, confidence interval; OR, odds ratio.

Table 2 describes the association between AAM and the risk of macrosomia. In the crude model, women with an AAM of less than 13 years exhibited a significantly increased risk of macrosomia compared to those with an AAM between 13 and 15 years (OR: 1.89, 95% CI: 1.38–2.58, *P* trend: 0.019). This association remained after adjusting for age, pre-pregnancy BMI, parity, timing of delivery, menstrual cycle, menses length, regular menstruation, and dysmenorrhea, (OR: 1.86, 95% CI: 1.35–2.57, *P* trend: 0.034). After further adjustment for educational attainment, employment status, monthly incomes, ethnicity, current smoking status, exposure to second-hand smoke, alcohol consumption, physical activity, total energy intake, nutritional supplements usage, and dietary patterns, women with an AAM of less than 13 years continued to exhibit a 90% increased risk of delivering a macrosomia infant (OR: 1.90, 95% CI: 1.37–2.62, *P* trend: 0.042). RCS analysis indicated a non-linear relationship between AAM and the risk of macrosomia (*P* for overall =0.0430, *P* for nonlinear =0.040) (Figure 1). Subgroup analysis indicated that the correlation between AAM and macrosomia remained largely consistent across different strata (Figure 2).

**Table 2:** Associations between the age at menarche and macrosomia risk (*n* = 2,554)^a^.

Model	Maternal age at menarche (year)			*P*-trend
	13–15	<13	>15	
Case/total	77/1,433	93/961	10/160	-
Model 1	1.00 (reference.)	1.89 (1.38, 2.58)	1.17 (0.60, 2.32)	0.019
Model 2	1.00 (reference.)	1.86 (1.35, 2.57)	1.24 (0.62, 2.48)	0.034
Model 3	1.00 (reference.)	1.90 (1.37, 2.62)	1.32 (0.66, 2.62)	0.042

^a^ Values are odds ratios (95% confidence intervals) estimated by binary logistic models.

Model 1: unadjusted.

Model 2: adjusted for age, pre-pregnancy body mass index, parity, timing of delivery, menstrual cycle, menses length, regular menstruation, dysmenorrhea.

Model 3 was further adjusted for educational attainment, employment status, monthly incomes, ethnicity, current smoking status, exposure to second-hand smoke, alcohol consumption, physical activity, total energy intake, nutritional supplements usage, and dietary patterns.

Dietary patterns included “meat, shrimp and fish” dietary pattern; “vegetables, soybean and soybean products” dietary pattern and “animal offal, processed meat and baked food” dietary pattern.

### 3.3 Association between dietary patterns and macrosomia

Table 3 outlines the associations between dietary patterns and the risk of macrosomia. After full adjustment, women adhering to the “meat, shrimp, and fish” dietary pattern, with scores above the median, exhibited a reduced risk of macrosomia when compared to those with scores below the median (OR: 0.64, 95% CI: 0.46–0.89). Similarly, those following the “vegetables, soybeans, and soybean products” dietary pattern and scoring above the median demonstrated a 52% reduction in the risk of macrosomia (OR: 0.48, 95% CI: 0.33–0.70).

**Table 3:** Associations between the dietary patterns and macrosomia risk (*n* = 2,554)^a^.

Dietary patterns	Below median	Above median
Meat, shrimp and fish dietary pattern		
Case/*n*	109/1,277	71/1,277
Model 1	1.00 (reference.)	0.66 (0.48, 0.90)
Model 2	1.00 (reference.)	0.65 (0.41, 0.78)
Model 3	1.00 (reference.)	0.64 (0.46, 0.89)
Vegetables, soybean and soybean products dietary pattern		
Case/n	112/1,277	68/1,277
Model 1	1.00 (reference.)	0.59 (0.43, 0.80)
Model 2	1.00 (reference.)	0.56 (0.41, 0.78)
Model 3	1.00 (reference.)	0.48 (0.33, 0.70)
Animal offal, processed meat and baked food dietary pattern		
Case/n	79/1,277	101/1,277
Model 1	1.00 (reference.)	1.31 (0.96, 1.78)
Model 2	1.00 (reference.)	1.41 (1.03, 1.93)
Model 3	1.00 (reference.)	1.34 (0.97, 1.86)

^a^ Values are odds ratios (95% confidence intervals) estimated by binary logistic models.

Model 1: unadjusted.

Model 2: adjusted for age, age at menarche, pre-pregnancy body mass index, parity, timing of delivery, menstrual cycle, menses length, regular menstruation, dysmenorrhea.

Model 3 was further adjusted for educational attainment, employment status, monthly incomes, ethnicity, current smoking, exposure to second-hand smoke, alcohol consumption, physical activity, total energy intake, nutritional supplements usage, and dietary patterns.

Dietary patterns included “meat, shrimp and fish” dietary pattern; “vegetables, soybean and soybean products” dietary pattern and “animal offal, processed meat and baked food” dietary pattern.

### 3.4 The modified effect of dietary patterns

Table 4 presents the joint associations of AAM and dietary patterns with the risk of macrosomia. In the “meat, shrimp, and fish” dietary pattern, the reduced risk of macrosomia was observed among pregnant women with an AAM below 13 years who scored above the median (OR: 1.45, 95% CI: 0.88–2.40), in contrast to those with lower dietary pattern scores (OR: 2.33, 95% CI: 1.51–3.59). Similarly, within the same AAM subgroup, an inverse association was identified between higher adherence to the “vegetables, soybeans, and soybean products” dietary pattern and macrosomia risk (OR: 1.78, 95% CI: 1.04–3.07) compared to lower adherence (OR: 2.02, 95% CI: 1.34–3.05). Conversely, for the “animal offal, processed meat, and baked food” dietary pattern, a 61% increased risk of macrosomia was demonstrated in the higher-scoring group (OR: 2.23, 95% CI: 1.42–3.50) relative to the lower-scoring group (OR: 1.62, 95% CI: 1.01–2.61) among women with an AAM below 13 years.

**Table 4:** Joint associations of age at menarche and dietary pattern with fetal macrosomia risk (*n* = 2,554)^a^.

Dietary Patterns	Age at menarche			*P* for interaction
	13–15	<13	>15	
“Meat, shrimp and fish” dietary pattern				0.094
Below median	1.00 (reference.)	2.33 (1.51, 3.59)	2.12 (0.96, 4.69)	
Above median	1.00 (reference.)	1.45 (0.88, 2.40)	0.27 (0.04, 2.08)	
“Vegetables, soybean and soybean products” dietary pattern				0.158
Below median	1.00 (reference.)	2.02 (1.34, 3.05)	0.59 (0.17, 1.99)	
Above median	1.00 (reference.)	1.78 (1.04, 3.07)	2.27 (0.91, 5.67)	
“Animal offal, processed meat and baked food” dietary pattern				0.612
Below median	1.00 (reference.)	1.62 (1.01, 2.61)	1.89 (0.87, 4.04)	
Above median	1.00 (reference.)	2.23 (1.42, 3.50)	2.48 (1.13, 5.40)	

^a^ Values are odds ratios (95% confidence intervals) estimated by binary logistic models.

Model 1: unadjusted.

Model 2: adjusted for age, pre-pregnancy body mass index, parity, timing of delivery, menstrual cycle, menses length, regular menstruation, dysmenorrhea.

Mode 3: further adjusted for educational attainment, employment status, monthly incomes, ethnicity, current smoking, exposure to second-hand smoke, alcohol consumption, physical activity, total energy intake, nutritional supplements usage, and other dietary patterns.

Dietary patterns included “meat, shrimp and fish” dietary pattern; “vegetables, soybean and soybean products” dietary pattern and “animal offal, processed meat and baked food” dietary pattern.

### 3.5 Sensitivity analysis

In sensitivity analyses, the association between AAM and macrosomia risk was evaluated after excluding participants who delivered preterm offspring (gestational age < 37 weeks; *n* = 2343). Following adjustment for all covariates, AAM < 13 years remains significantly associated with an increased risk of macrosomia (OR: 1.91, 95% CI: 1.38–2.65; *P* for trend: 0.041) (Supplementary Table 5). Further stratification by the three dietary patterns reaffirmed the robustness of this association (Supplementary Table 6).

## 4 Discussion

In this prospective cohort study, we found that pregnant women with AAM < 13 years had a higher risk of macrosomia after adjusting for potential confounders. This association was modified by dietary pattern; the elevated risk was attenuated among women with healthier diets and amplified among those following less healthy diets. To our knowledge, this is the first large-scale prospective cohort study to identify maternal AAM as a novel risk factor for macrosomia and to demonstrate that its association with macrosomia is modifiable by dietary habits. These findings provide new perspectives on the etiology and primary prevention of macrosomia, highlighting the potential for dietary habits to mitigate risk, particularly in women who experienced AAM before 13 years old.

Our results were supported by Reshetnikova et al. (13), investigated the association between AAM-related SNPs and neonatal birth weight from 716 mother-newborn pairs. They found that maternal genetic predisposition to early AAM increased the birth weight through maternal metabolic dysregulation. While previous research has established a link between AAM and certain metabolic outcomes, its specific association with macrosomia remains unknown, and the limited sample size may constrain the validity and generalizability of the conclusions. In the large-scale prospective cohort study, we demonstrated that AAM < 13 years old is significantly associated with higher risk of macrosomia. Furthermore, we identified adherence to a healthy dietary pattern (characterized by high intake of vegetables and soy products) reduced the association; while an unhealthy pattern rich in processed meats and baked goods was associated with higher risk. This study elucidates the relationship between AAM and macrosomia and provides new evidence for the potential role of dietary habits in mitigating this risk.

RCS analysis revealed a significant U-shaped non-linear relationship between maternal AAM and the risk of offspring macrosomia. Specifically, the odds of macrosomia were markedly elevated among women with early menarche, declined to the lowest level at the reference age, and then showed a positive trend of increasing risk with AAM values greater than 15 years, although this association did not reach statistical significance at the individual later age points. This U-shaped pattern suggests that both ends of the AAM spectrum, particularly early menarche, may be independent markers of elevated macrosomia risk in offspring, while later menarche appears to be associated with a directional increase in risk that warrants further investigation. From a public health perspective, the identification of this non-linear relationship reinforces the value of screening for AAM in prenatal care. Women with very early menarche may represent a high-risk subgroup warranting targeted antenatal monitoring. The observed positive trend among women with AAM >15 years also suggests that closer attention may be beneficial for this group, although further research is needed to confirm the clinical significance of this association.

The precise mechanisms linking early AAM to macrosomia risk remain incompletely understood; however, several potential pathways have been proposed, including hormonal changes, metabolic dysregulation, and inflammatory processes. Early menarche has been linked to an elevated risk of insulin resistance and diabetes mellitus in adult life, potentially influenced by nutritional and environmental factors (40). Maternal hyperglycemia facilitates increased fetus glucose transfer across the placenta, prompting the fetal pancreas to secrete more insulin (41, 42), function as a growth factor, fosters excessive fetal growth and development (43). Therefore, controlling maternal blood glucose levels and regulating fetal insulin secretion during pregnancy are recognized as pivotal strategies for preventing macrosomia. Animal studies have revealed that administering composite dietary fiber supplements during gestation improves insulin sensitivity (44). Furthermore, a large-scale study in the US which involving 24,182 individuals, found that dietary patterns rich in vitamins, minerals, and fiber are linked to delayed gastric emptying, which slows digestion and glucose absorption, thereby reducing plasma insulin levels and enhancing glucose tolerance (45). It is supposed that vegetables rich in dietary fiber may suppress appetite and reduce caloric intake, thereby improving insulin sensitivity (46, 47), potentially lowering the risk of macrosomia in pregnant women who experienced earlier menarche. In our study, we observed that a dietary pattern characterized by high consumption of vegetables, soybeans, and soybean products was associated with a lower risk of macrosomia. We propose that this effect may stem from the ability of vegetable- and fruit-rich foods to mitigate the risk of macrosomia associated with early AAM by modulating insulin resistance and ameliorating hyperglycemic conditions.

Furthermore, earlier menarche has been correlated with a greater prevalence of hypertriglyceridemia (48, 49), which is a well-established predictor of macrosomia (22). Throughout pregnancy, the placenta expresses lipoprotein receptors, fatty acid-binding proteins, and lipases, which collectively facilitate the hydrolysis of maternal triglycerides. This process results in an excessive transplacental transfer of free fatty acids to the fetus (50), implicated in the development of macrosomia (22). Furthermore, limited placental transfer of glycerol—a key substrate for maternal gluconeogenesis—promotes increased maternal to fetal glucose transfer, establishing a metabolic milieu that facilitates fetal overgrowth and macrosomia (22). Therefore, intervening to regulate the elevated triglyceride levels linked to earlier menarche could serve as a promising approach to curtail the risk of fetal macrosomia. Notably, evidence indicates that α-linolenic acid (ALA), a plant-based n-3 fatty acid that is richly found in vegetables, soybeans, and soybean products (51), can effectively suppress the hepatic synthesis of very low-density lipoprotein cholesterol and triglycerides (52), consequently playing a role in lowering serum triglyceride concentrations (53, 54). And the vitamins, minerals, and dietary fiber abundant in plant-based foods act as vital sources of unsaturated fatty acids, flavonoids, bioactive peptides, and phytosterols. These compounds synergistically optimize the regulation of serum triglyceride levels (55, 56), finally diminishing the probability of macrosomia occurrence (22, 53).

Inflammation has been posited as a pivotal mediator. Early menarche extends the duration of estrogen exposure, via hormone receptor activation on immune cells (notably lymphocytes) (19), triggers inflammatory pathways (20, 57). This activation prompts the release of pro-inflammatory cytokines, such as interleukin-6 (IL-6) and tumor necrosis factor-α (TNF-α), which have been shown to inhibit adiponectin synthesis (58), consequently heightening the risk of macrosomia (59). The pro-inflammatory milieu induced by early estrogen exposure may be further intensified by suboptimal dietary habits, exacerbating the risk of macrosomia. Diets rich in processed meats, baked goods, and other industrialized food products, characterized by high levels of saturated and trans fats, as well as added sugars contribute to a systemic pro-inflammatory state (60). Excessive intake of these foods disrupts the pregnant women redox balance (61–63), leading to maternal low-grade inflammation and fostering an inflammatory intrauterine environment, in turn, impairing normal placental function. This dysfunction promotes the release of free fatty acids and glucose to the fetus, adversely impacting its growth and development, and ultimately increasing the likelihood of macrosomia (64).

Base on a large-scale population cohort, this study offers prospective evidence to substantiate the hypothesized link between AAM and the risk of macrosomia. Furthermore, as the first major contribution to the existing body of research on the relationship between menarche and macrosomia, this cohort study also sheds light on the impact of dietary patterns on this association. Several limitations merit consideration in this study. Firstly, data regarding AAM were obtained through self-reports from pregnant women, which may introduce potential recall bias. Nevertheless, previous studies have consistently demonstrated a strong correlation between self-reported AAM in adulthood and actual AAM (65). Secondly, dietary intake was evaluated using the FFQ designed to capture dietary habits during the year preceding pregnancy, which may introduce bias in estimating actual intake during the pregnancy period. However, research has indicated that dietary intake measured at a fixed point before or during pregnancy can serve as a reliable indicator of overall dietary patterns throughout pregnancy (33, 66). Thirdly, the dietary patterns derived through principal component analysis account for only a proportion of the total variance in food groups, thus representing the optimal model based solely on the explainable variance. Fourthly, the preparation and cooking method of food may influence dietary intake. Fifthly, due to resource limitations, our analysis did not incorporate GDM and gestational weight gain, which may introduce bias into the observed findings (67). Previous studies have indicated that GDM is associated with both AAM and macrosomia, with ORs of 1.08 (68) and 1.70 (69) respectively. Nevertheless, the E-values analysis showed that the cumulative impact of the two ORs remained within the limits of the E value (*E* = 3.21), suggesting that the association between early AAM and macrosomia risk cannot be entirely attributed to GDM. Similarly, the relative risk of gestational weight gain associated with menarche was calculated to be 2.35 (70), which was demonstrated to be inadequate for fully elucidating the relationship between early menarche and the risk of macrosomia. Sixthly, despite adjustments for multiple confounding factors, due to the observational design, we cannot completely exclude the possibility of residual bias. Our study cohort was composed of Chinese pregnant women, which may limit the generalizability of our results to other demographic groups. Consequently, larger-scale, multicenter investigations are essential to confirm and extend our findings.

## 5 Conclusions

In conclusion, our study reveals that pregnant women experiencing early AAM face an elevated risk of giving birth to macrosomia. However, this risk can potentially be mitigated through adherence to a healthier dietary regimen, whereas unhealthy dietary patterns may further augment it. Our findings provide novel perspectives on the pathogenesis of macrosomia and highlight the critical role of healthy dietary habits.

## Data Availability

The original contributions presented in the study are included in the article/Supplementary material, further inquiries can be directed to the corresponding authors.
