# Supplementary material for: Association of menarche age with macrosomia and modified effect from dietary pattern: findings from the Chinese pregnant women
Source: Front Nutr. 2026 Apr 2;13:1777526. doi: 10.3389/fnut.2026.1777526 (PMC13082937; doi:10.3389/fnut.2026.1777526)
Supplement: Supplementary file 1 [file Supplementary_file_1.docx]

**Additional file 1**

**Supplemental Figure S1.** Flow diagram for selection of participants in the study.

**Supplemental Table S1.** Baseline characteristics of the pregnant women according to macrosomia.

**Supplemental Table S2**. Food entries included in the 23 food groups included in the factor analysis.

**Supplemental Figure S2.** The radar chart depicts three distinct dietary patterns.

**Supplemental Figure S3.** A directed acyclic graph illustrates the potential covariates between age at menarche and macrosomia.

**Supplemental Table S3**. Factor eigenvalues, variance contributions and cumulative variance contributions.

**Supplemental Table S4**. Dietary pattern factor loading.

**Supplemental Table S5.** Associations between the age at menarche and macrosomia risk, excluding those with low birth weight (<2500g) and women with premature offspring. (n=2,343).

**Supplemental Table S6.** Associations between the age at menarche and macrosomia risk stratified by dietary pattern, excluding those with low birth weight (<2500g) and women with premature offspring. (n=2,343).


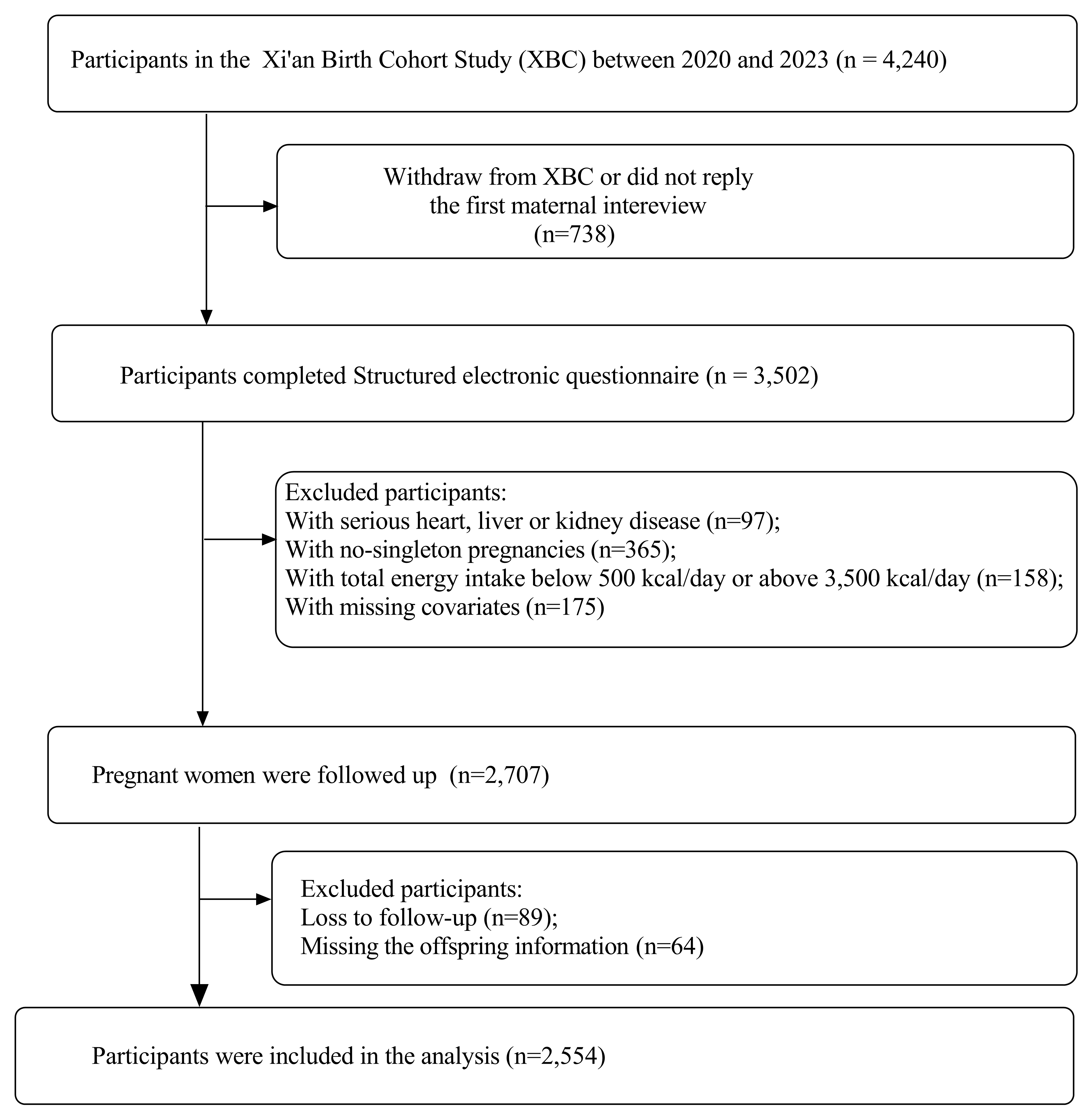


**Supplemental Figure. S1.** Flow diagram for selection of participants in the study.

**Supplemental Table 1**. Food entries included in the 23 food groups included in the factor analysis.

| No. | Food Groups | Food items |
| --- | --- | --- |
| 1 | Beef | Beef |
| 2 | Freshwater fish | Crucian, carp |
| 3 | Shrimp | Shrimp |
| 4 | Saltwater fish | Yellow croaker, hairtail |
| 5 | Chicken | Chicken |
| 6 | Mutton | Mutton |
| 7 | Yogurt | Yogurt |
| 8 | Pork | Pork |
| 9 | Milk | Milk |
| 10 | Egg | Egg |
| 11 | Vegetables | Spinach, leek, rape, cabbages, baby bok choy, chrysanthemum coronariums, lettuces, celery, asparagus lettuce, bamboo shoots, wild rice stems, purple cabbage, cauliflowers, broccoli/mater convolvulus/amaranth,  Lotus roots, tomatoes, fresh peppers、Potatoes, sweet potatoes, cucumbers, pumpkins, wax gourd, etc |
| 12 | Soybean and soybean products | Bean sprouts (yellow bean sprout and mung bean sprout), soybeans, black soybeans, tofu, shredded tofu/tofu skin/dried tofu/dried bean curd sticks, soy milk |
| 13 | Ginger | Ginger |
| 14 | Nuts | Peanuts, walnuts and sunflower seeds |
| 15 | Fruits | Pears, apples, peaches, plums, oranges, tangerines, grapefruit, mangoes, persimmons, papayas, bananas, watermelons, muskmelon, Hami melon, pineapples, etc |
| 16 | Dried peppers | Dried peppers |
| 17 | Rice and noodles | Rice, rice flour/rice noodles, noodles, steamed buns/steamed twisted roll, steamed stuffed bun, dumplings |
| 18 | Corns | Corns |
| 19 | Animal offal | Pork intestines |
| 20 | Processed meat | Bacon, sausages, ham sausages |
| 21 | Baked food | Biscuits, cakes, bread, Chinese snacks (green bean cake) |
| 22 | Sugary drinks | Coke, sprite, coffee, ground coffee, other sugary beverages, such as iced peaks, orange juice, milk tea |
| 23 | Deep-fried dough sticks | Deep-fried dough sticks |

**Supplemental Table 2**. Factor eigenvalues, variance contributions and cumulative variance contributions.

| Factor | Eigenvalues | Variance contributions (%) | Cumulative Contribution (%) |
| --- | --- | --- | --- |
| “Meat, shrimp and fish” dietary pattern | 3.438 | 12.728 | 12.728 |
| “Vegetables, soybean and soybean” products dietary pattern | 1.707 | 8.276 | 21.005 |
| “Animal offal, processed meat and baked food” dietary pattern | 1.510 | 7.930 | 28.935 |

**Supplemental Table 3.** Dietary pattern factor loading.

| Food groups | Dietary pattern | | |
| --- | --- | --- | --- |
|  | "Meat, shrimp and fish”  dietary pattern | “Vegetables, soybean and  soybean products” dietary pattern | “Animal offal, processed meat and baked food” dietary pattern |
| Beef | 0.656 | 0.138 | 0.140 |
| freshwater fish | 0.646 | -0.055 | 0.043 |
| Shrimp | 0.638 | -0.090 | 0.120 |
| Saltwater fish | 0.587 | -0.113 | 0.188 |
| Chicken | 0.462 | 0.081 | 0.297 |
| Mutton | 0.445 | -0.069 | 0.342 |
| Yogurt | 0.389 | 0.210 | -0.049 |
| Pork | 0.358 | 0.107 | -0.034 |
| Milk | 0.350 | 0.136 | -0.279 |
| Egg | 0.341 | 0.329 | -0.281 |
| Vegetables | 0.231 | 0.676 | 0.147 |
| Soybean and soybean products | 0.196 | 0.588 | -0.068 |
| Ginger | -0.079 | 0.457 | 0.141 |
| Nuts | 0.307 | 0.417 | -0.001 |
| Fruits | 0.275 | 0.362 | -0.036 |
| Dried peppers | -0.135 | 0.361 | 0.276 |
| Rice and noodles | -0.099 | 0.326 | 0.110 |
| Corn | -0.005 | 0.303 | -0.063 |
| Animal offal | 0.259 | 0.037 | 0.603 |
| Processed meat | 0.113 | 0.009 | 0.601 |
| Baked food | 0.055 | 0.037 | 0.500 |
| Sugary drinks | 0.098 | 0.044 | 0.480 |
| Deep-fried dough sticks | -0.107 | 0.131 | 0.209 |


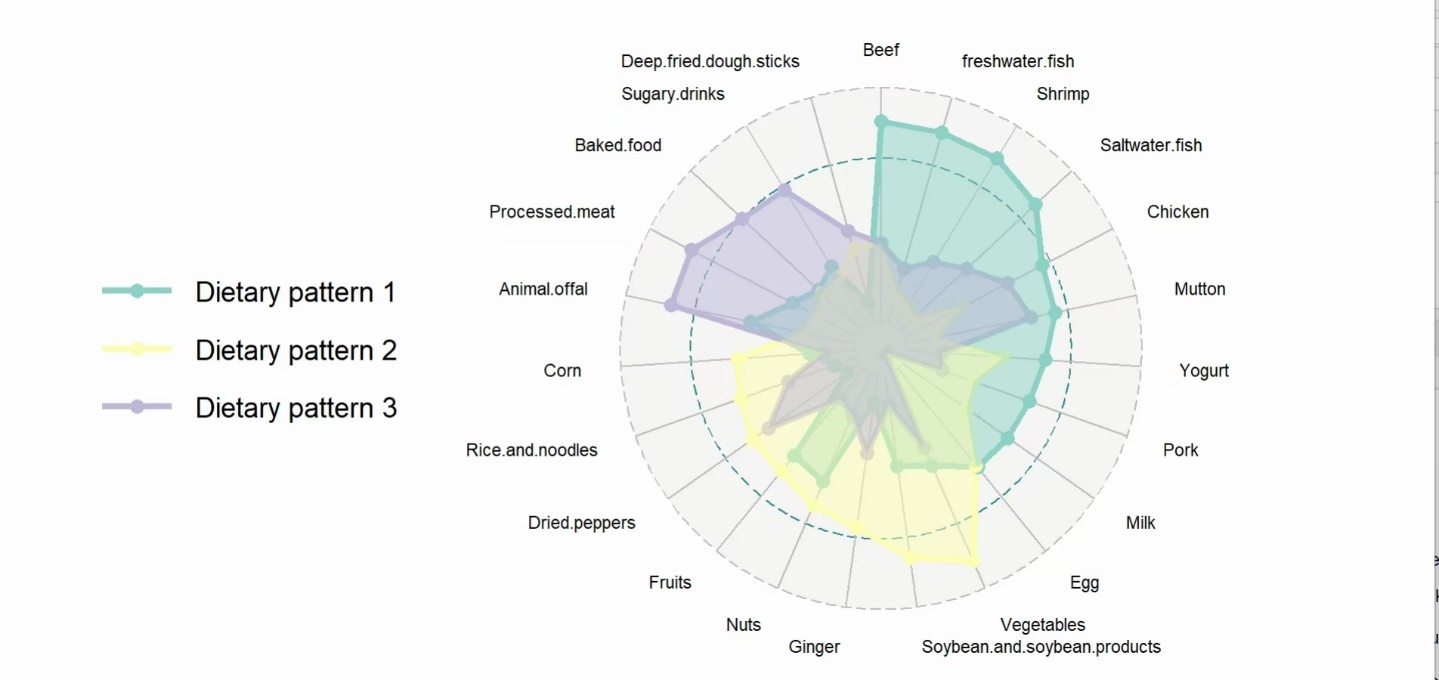
 **Supplemental Figure 2.** The radar chart depicts three distinct dietary patterns. At the chart's center, the origin of consumption is denoted, with the endpoints of radial lines indicating the maximum potential consumption levels for various food categories within specific dietary patterns. Dietary pattern 1, represented by light green, is characterized as the meat, shrimp, and fish dietary pattern, primarily consisting of beef, freshwater fish, shrimp and saltwater fish. Dietary pattern 2, denoted by light yellow, is designated as the vegetables, soybean and soybean products dietary pattern, dominated by vegetables, soybeans and soybean products, ginger, and nuts. Dietary pattern 3, indicated by light purple, is termed the animal offal, processed meat, and baked food dietary pattern, primarily encompassing animal offal, processed meat, baked food, sugary drinks, and deep-fried dough sticks.

**
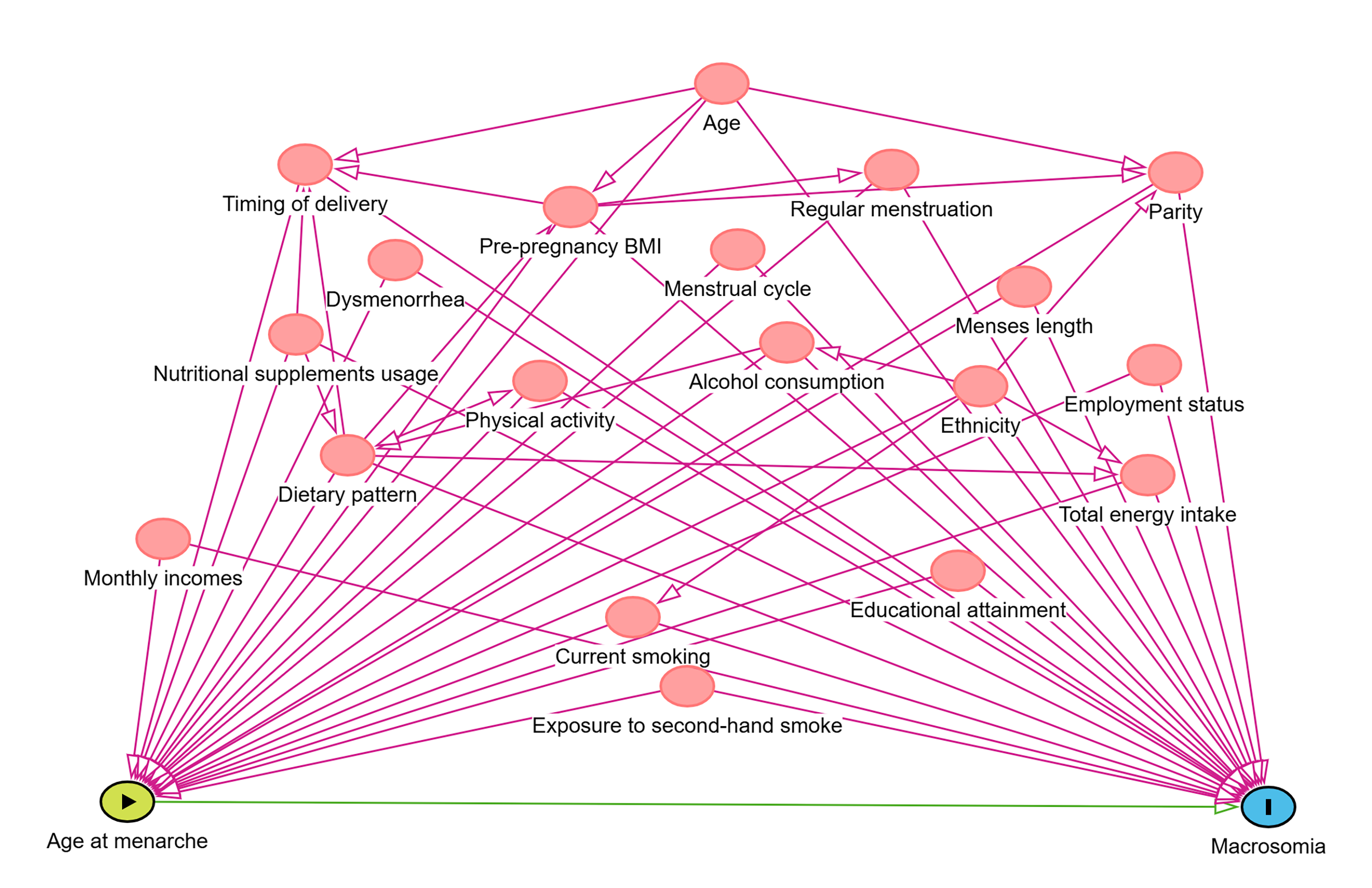
**

**Supplemental Figure 3.** A directed acyclic graph illustrates the potential covariates between age at menarche and macrosomia. In the graphical representation, solid rose-pink circles denote shared ancestral factors between exposure and outcome. Light green solid circles marked with black triangles represent exposure factors. Concurrently, light blue solid circles, adorned with black rectangles, indicate outcome factors. Dark green arrows are employed to delineate causal pathways, whereas rose-pink arrows are utilized to illustrate bias pathways. The identified confounding factors included age, educational attainment, employment status, monthly incomes, ethnicity, current smoking status, exposure to second-hand smoke, alcohol consumption, physical activity, parity, timing of delivery, menstrual cycle, menses length, regular menstruation, dysmenorrhea and pre-pregnancy BMI, total energy intake, nutritional supplements usage and dietary patterns. Abbreviations: BMI: body mass index;。

**Supplemental Table 4.** Baseline characteristics of the pregnant women according to macrosomia

| Characteristics | No- macrosomia (n=2374) | Macrosomia (n=180) |
| --- | --- | --- |
| Number of participants | 2374 | 180 |
| Age (years) | 30.0 (28.0, 32.0) | 30.0 (28.0, 33.0) |
| Educational attainment (under college) | 311 (13.1) | 30 (16.7) |
| Employment status (no) | 767 (32.3) | 64 (35.6) |
| Monthly incomes (≤9000 CNY) | 2305 (97.1) | 178 (98.9) |
| Ethnicity (minority) | 25 (1.1) | 0 (0) |
| Current smoking | 285 (12.0) | 27 (15.0) |
| Exposure to second-hand smoke | 31 (1.3) | 2 (1.1) |
| Alcohol consumption | 13 (0.5) | 3 (1.7) |
| Physical activity (MET-min/week) | 600.0 (462.0, 1146.0) | 684.0 (462.0, 1659.0) |
| Pre-pregnancy BMI (kg/m^2^) |  |  |
| <18.5 | 291 (12.3) | 14 (7.8) |
| 18.5-24.0 | 1656 (69.8) | 125 (69.4) |
| ≥24.0 | 427 (18.0) | 41 (22.8) |
| Parity (Primigravida) | 1128 (47.5) | 76 (42.2) |
| Timing of delivery (weeks) | 39 (38, 40) | 40 (39, 40) |
| Menstrual cycle (d) | 29.0 (28.0, 30.0) | 30.0 (28.0, 31.0) |
| Menses length (d) | 5 (5, 6) | 5 (4, 6) |
| Regular menstruation (no) | 158 (6.7) | 15 (8.3) |
| Dysmenorrhea | 1151 (48.5) | 84 (46.7) |
| Total energy intake (kcal/d) | 2360.1 (1933.2, 2781.0) | 2265.0 (1884.4, 2714.0) |
| Nutritional supplements usage (no) | 117 (4.9) | 7 (3.9) |
| Age at menarche |  |  |
| <13 | 1356 (57.1) | 93 (51.7) |
| 13-15 | 868 (36.6) | 77 (42.8) |
| >15 | 150 (6.3) | 10 (5.6) |

Continuous variables were expressed as median (interquartile range) and categorical variables as n (%). Abbreviations: BMI: body mass index; MET: metabolic equivalent.

**Supplemental Table 5.** Associations between the age at menarche and macrosomia risk, excluding those with low birth weight (<2500g) and women with premature offspring. (n=2,343) ^a^

|  | Maternal age at menarche (year) | | | | | *P*-trend |
| --- | --- | --- | --- | --- | --- | --- |
|  | 13-15 | | <13 | | > 15 |  |
| Case, n (%) | 77/1433 | 93/961 | | 10 /160 | | - |
| Model 1 | 1.00 (reference.) | 1.94 (1.41, 2.65) | | 1.20 (0.61, 2.37) | | 0.017 |
| Model 2 | 1.00 (reference.) | 1.88 (1.36, 2.59) | | 1.29 (0.65, 2.58) | | 0.038 |
| Model 3 | 1.00 (reference.) | 1.91 (1.38, 2.65) | | 1.34 (0.67, 2.71) | | 0.041 |

^a^ Values are odds ratios (95% confidence intervals) estimated by binary logistic models.

Model 1: unadjusted.

Model 2: adjusted for age, pre-pregnancy body mass index, parity, timing of delivery, menstrual cycle, menses length, regular menstruation, dysmenorrhea.

Model 3 was further adjusted for educational attainment, employment status, monthly incomes, ethnicity, current smoking, exposure to second-hand smoke, alcohol consumption, physical activity, total energy intake, nutritional supplements usage and dietary pattern.

Dietary pattern included “meat, shrimp and fish” dietary pattern, “vegetables, soybean and soybean products” dietary pattern and “animal offal, processed meat and baked food” dietary pattern.

**Supplemental Table 6.** Associations between the age at menarche and macrosomia risk stratified by dietary pattern, excluding those with low birth weight (<2500g) and women with premature offspring. (n=2,343) ^a^

|  | Age at menarche (years) | | | *P* for interaction |
| --- | --- | --- | --- | --- |
|  | 13-15 | <13 | >15 |  |
| “Meat, shrimp and fish” dietary pattern |  |  |  | 0.283 |
| Below median | 1.00 (reference.) | 2.33 (1.51, 3.61) | 2.16 (0.97, 4.81) |  |
| Above median | 1.00 (reference.) | 1.48 (0.89, 2.46) | 0.28 (0.04, 2.09) |  |
| “Vegetables, soybean and soybean products” dietary pattern |  |  |  | 0.172 |
| Below median | 1.00 (reference.) | 2.02 (1.33, 3.06) | 0.58 (0.17, 1.98) |  |
| Above median | 1.00 (reference.) | 1.83 (1.06, 3.15) | 2.28 (0.91, 5.68) |  |
| “Animal offal, processed meat and baked food” dietary pattern |  |  |  | 0.631 |
| Below median | 1.00 (reference.) | 1.65 (1.03, 2.65) | NA ^b^ |  |
| Above median | 1.00 (reference.) | 2.24 (1.42, 3.54) | 2.48 (1.13, 5.44) |  |

^a^ Values are odds ratios (95% confidence intervals) estimated by binary logistic models.

^b^ OR (95%CI) was not obtained due to the small sample size and no cases in this subgroup.

Model 1: unadjusted.

Model 2: adjusted for age, pre-pregnancy body mass index, parity, timing of delivery, menstrual cycle, menses length, regular menstruation, dysmenorrhea.

Model 3 was further adjusted for educational attainment, employment status, monthly incomes, ethnicity, current smoking, exposure to second-hand smoke, alcohol consumption, physical activity, total energy intake, nutritional supplements usage and other dietary patterns.

Dietary pattern included “meat, shrimp and fish” dietary pattern, “vegetables, soybean and soybean products” dietary pattern and “animal offal, processed meat and baked food” dietary pattern.
